# Self-Assembly of Block Copolymers

**DOI:** 10.3390/polym12040794

**Published:** 2020-04-02

**Authors:** Volker Abetz

**Affiliations:** 1 Institute of Physical Chemistry, Universität Hamburg, Martin-Luther-King-Platz 6, 20146 Hamburg, Germany; volker.abetz@hzg.de; Tel.: +49-4152-87-2461; 2 Helmholtz-Zentrum Geesthacht, Institute of Polymer Research, Max-Planck-Straße 1, 21502 Geesthacht, Germany

Block copolymers and block-copolymer-containing blends represent a fascinating class of soft matter and can self-assemble in a variety of ordered structures on the mesoscale [1,2,3,4,5,6]. A requirement for block copolymer self-assembly is a segmental incompatibility between the chemically different blocks, which can also be connected with each other in many different topologies. The selective interactions of these blocks with other polymers or solvents further increases the number of possible, and sometimes even hierarchical, structures [4,6,7,8]. Especially, tailor-made block copolymers are of interest, which can be obtained by living or controlled polymerization techniques. While living anionic polymerization has been applied for a long time to this goal [9], controlled radical polymerization techniques in particular have become more and more popular during the last few decades, as they require less rigorous purity of solvents and monomers [10]. The experimental developments also stimulated theoretical investigations of the block copolymer self-assembly, which led to fundamental concepts of the morphological properties of these materials, such as the weak segregation [1,11] and the strong segregation theories [12,13]. Due to their long chain structure, the kinetics of the structure formation is an important issue, and many non-equilibrium or meta-stable states can be frozen in, which makes processing a tool to manipulate both the structure and properties of these materials [14]. With increasing computer power, computer simulations have also become more and more relevant for the understanding of the microphase formation of these macromolecules [15].

In this Special Issue on the self-assembly of block copolymers, a collection of manuscripts from all over the globe deal with some of the above-mentioned aspects. The topics of these manuscripts are summarized in Figure 1.

The team of Jianbo Tan investigates light-induced controlled radical polymerization of an amphiphilic diblock copolymer starting from hydrophilic macro-RAFT agents, where the variation of the concentration of the second, hydrophobic monomer (benzyl methacrylate), the solvent mixture (binary mixture of water with different alcohols) and the temperature of polymerization are varied and lead to different micellar or vesicular structures during polymerization [16]. Labeesh Kumar et al. study the selectivity of different alcohols in the solution self-assembly of polystyrene-*block*-poly(4-vinylpyridine) diblock copolymers as a function of temperature and compare the results obtained by dynamic light scattering with transmission electron microscopy as well as the swelling behavior of the individual homopolymers studied by spectroscopic ellipsometry [17]. The use of micellar aggregates for carriage of drugs is investigated in several contributions to this Special Issue, as there is a big societal demand for improved treatments of illnesses such as diabetes or cancer. The group of Stergios Pispas presents a study on an amphiphilic triblock terpolymer composed of a cationic block, a hydrophobic block, and a hydrophilic, uncharged block, namely poly[quaternized 2-(dimethylamino)ethyl methacrylate]-*block*-poly(lauryl methacrylate)-*block*-poly[oligo(ethylene- glycol)], where the positively charged block can coordinate with the negative charges of insulin. The formed colloidal aggregates are characterized with dynamic light scattering, fluorescence spectroscopy, scanning force microscopy and zeta potential measurements. The insulin does not change its structure (and thus functionality) by the complexation [18]. Yebang Tan and his coworkers present an example of supramolecular chemistry by quaternizing the pyridine rings of poly(ethylene oxide)-*block*-poly(4-vinylpyridine) with 4-chloromethyl benzonitrile and using this diblock copolymer for preparation of pseudopolyrotoxane by anchoring cuburituril [7] at the functionalized side groups of the diblock copolymer. This leads to a micellar fluorescent system which can host drugs such as doxorubicin, which makes this system interesting for cancer theranostics [19]. Junting Jiang et al. present an interesting study on a poly(carboxybetaine methacrylate)-*block*-polycaprolactone-*block*-poly(carboxybetaine methacrylate) triblock copolymer obtained by RAFT polymerization from the α-ω functionalized middle block. This triblock copolymer can also host and deliver doxorubicin and thus may be an interesting candidate for cancer chemotherapeutics [20]. Multicompartment nanogels of poly(ethylene oxide)-*block*-poly(propylene oxide)-*block*-poly(ethylene oxide) (PEO-*b*-PPO-*b*-PEO) grafted onto gelatin, where several hydrophobic PPO microdomains are embedded in a hydrophilic matrix of PEO and positively charged gelatin. These nanogels can host negatively charged, hydrophobic curcumin and display promising anti-cancer properties, as reported by Dinh Trung Nguyen et al. [21]. Not only drugs but also other guest molecules can be hosted by block copolymers. Sandra Rodríguez-Fabià et al. compare the uptake of carbon dioxide by an aqueous solution of monoethanolamine with a similar solution containing, in addition, a large amount of a PEO-*b*-PPO-*b*-PEO triblock copolymer which forms a lyotropic phase. Depending on the amount of CO_2_, the system shows a transition from a lamellar to a hexagonal phase due to an increased swelling of the PEO domains by an increase in the polarity of the mixed solvent [22]. There are many ways to use block copolymers for templating inorganic materials by using their self-assembled structures for hosting the precursors. Nina Yan et al. use spherical micelles of polystyrene-*block*-poly(4-vinylpyridine) with a PS core to first bind iron ions in the P4VP shell, which was then followed by atomic vapor deposition of titania. A final calcination step removes all organic components and transformed the iron ions of the inner shell into iron oxide, leading to inorganic hollow double-shell nanospheres [23]. A similar approach is demonstrated on vapor-annealed thin films of a PS-*b*-PEO diblock copolymer by Jin Xu et al. [24], where the cylindrical PEO domains are loaded with iron and cobalt ions, followed by an oxidative and thermal treatment to yield regularly arranged, ferrimagnetic cobalt ferrite nanodots with suitable transition temperature to superparamagnetic nanodots, making them interesting candidates for memory storage applications. A systematic study of vapor annealing to equilibrate polystyrene-*block*-poly(2-vinylpyridine) diblock copolymer films and corresponding homopolymers using in situ spectroscopic ellipsometry and different microscopies is presented by Xiao Cheng et al., who found the temperatures of the substrate and the solvent vapor to be main controlling parameters influencing the dynamics of equilibration [25]. Additionally, non-equilibrium structures obtained from block copolymer solution films can be of interest. Sarah Saleem et al. study the example of PS-*b*-P4VP-*b*-PSMA triblock terpolymers obtained by living anionic polymerization and the corresponding PS-*b*-P4VP-*b*-PGMA obtained by hydrolysis of the third block. In this study, the influence of the third block, either a non-polar poly(solketal methacrylate ) (PSMA) or a hydrophilic poly(glyceryl methacrylate) (PGMA), on the formation of an integral asymmetric membrane structure by non-solvent-induced phase separation is discussed [26]. Gordana Siljanovska Petreska et al. use RAFT mini-emulsion polymerization in water to obtain polystyrene-*block*-poly(ethylhexyl acrylate)-*block*-polystyrene thermoplastic elastomers, which show different morphologies and different thermomechanical properties when cast from solution and dried at room temperature or dried at a higher temperature, and which are another example of kinetically frozen-in structures [27]. Using free radical polymerization, J. Fage et al. synthesize poly(butyl acrylate)-*graft*-polystyrene graft copolymers [28]. They first copolymerize butyl acrylate with glycidyl acrylate or glycidyl methacrylate, followed by ring opening of the epoxy rings and connecting acrylic acid by esterification. In a second free radical polymerization, styrene is polymerized through the acrylic functions and free polystyrene is also generated simultaneously, thus leading to a blend, which shows promising mechanical and optical properties, which, however, are not as good as those of polybutadiene-based HIPS (high impact polystyrene) yet. Andrea Steinhaus et al. study blends of a well-defined polystyrene-*block*-polybutadiene-*block*-poly(methyl methacrylate) triblock terpolymer (PS-*b*-PB-*b*-PMMA) with various fractions of polybutadiene homopolymer PB [29]. The homopolymer swells the corresponding block of the triblock terpolymer, and with increasing amounts of PB the morphology can be changed from PB cylinders via perforated PB lamellae to PB lamellae, which can be crosslinked, and then isolated Janus objects are obtained after dissolving the material. The experimental observation by transmission electron microscopy is supported by dissipative particle dynamics (DPD) simulations. DPD simulations are also used by Alexey A. Gavrilov et al. to study the morphological phase behavior of a diblock copolymer consisting of an electrostatically charged and an uncharged block [30]. They compare the phase diagram of this block copolymer, which has purely electrostatic interactions, with a similar but uncharged block copolymer with a non-zero segmental interaction parameter and, surprisingly, find rather similar phase behavior, although the type of interaction is quite different. The authors conclude from this that introduction of electrical charges can already lead to microphase-separated structures for low degrees of polymerization. The final paper in this Special Issue deals with the theoretical prediction of novel block copolymer morphologies. Junham Cho applies field-theoretic simulations, which are based on self-consistent field theory, to describe novel co-continuous phases for diblock and triblock copolymers [31]. These structures are nice examples of the still-increasing and stimulating interaction between theory and experiment.

In summary, the manuscripts contributed to this Special Issue give some very nice insights into different ways to synthesize and process self-assembling block copolymers and some of their properties and possible applications, as well as some fundamental insights into their self-assembling behavior.

## Figures and Tables

**Figure 1:**
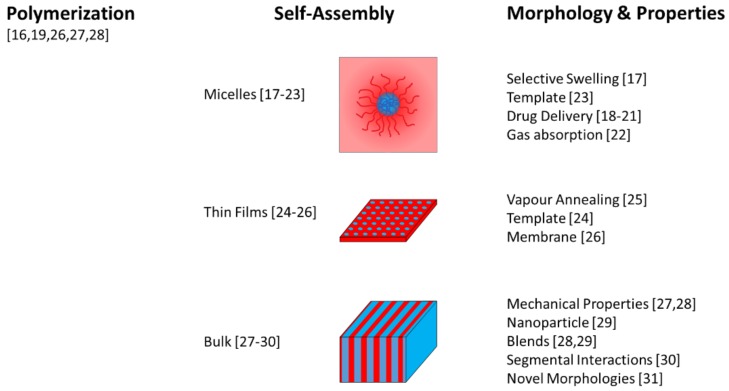
Overview of the topics covered by the manuscripts (indicated by the reference numbers) of this Special Issue.
